# Male breast MRI: a review of different pathological conditions

**DOI:** 10.1007/s11547-025-02084-x

**Published:** 2025-09-06

**Authors:** Marco Barillari, Piero Zanutto, Francesca Pellini, Elena Fiorio, Giulia Deguidi, Alessandra Invento, Alessia Nottegar, Mirko D’Onofrio, Giancarlo Mansueto

**Affiliations:** 1 Department of Diagnostic and Public Health, Section of Radiology, University of Verona, P.le L.A. Scuro 10, 37134 Verona, Italy; 2 Department of Radiology, Ospedale G.B. Rossi AOUI Verona, P.le L.A. Scuro 10, 37134 Verona, Italy; 3 Department of Breast Surgery, Ospedale Civile Maggiore AOUI Verona, P.le A. Stefani 1, 3716 Verona, Italy; 4 Department of Oncology, Ospedale Civile Maggiore AOUI Verona, P.le A. Stefani 1, 3716 Verona, Italy; 5 Department of Diagnostic and Public Health, Section of Pathology, University of Verona, P.le L.A. Scuro 10, 37134 Verona, Italy

**Keywords:** Male breast, MRI, Imaging, Breast cancer, Gynecomastia, Benign tumor

## Abstract

The male breast is predisposed to be affected by many of the same pathological processes as the female breast is. The diagnosis of male breast pathologies is generally achievable when clinical evaluation is combined with standard breast imaging methods such as mammography and ultrasound. Magnetic resonance imaging is also a valuable tool in diagnosing the main pathologies affecting the male breast, especially for evaluating pre- and post-surgical treatments and follow-up. However, although this technique has been sufficiently regulated and adopted by many breast radiologists for female breast imaging, its application in the diagnosis of male breast pathologies remains limited to a few specialized centers. This article, based on a retrospective analysis of the experience of the University of Verona, explores various aspects of male breast diseases, including benign conditions such as gynecomastia and breast implant ruptures in transgender women as well as malignant entities such as male breast cancer. Emphasis is placed on the distinctive morphological features, enhancement patterns and kinetics observed in male breast lesions on dynamic contrast-enhanced MRI. This article provides a comprehensive overview of the application of MRI in male breast disease assessment, highlighting the potential role of MRI as a complementary tool to traditional breast imaging techniques.

## Background

Over the past two decades, the percentage of men presenting with breast-related complaints has increased from 0.8 to 2.4%. Most symptomatic men are diagnosed with benign breast conditions such as gynecomastia or cutaneous lesions [1, 2]. Male breast cancer accounts for approximately 0.7% of all breast cancers [1] and 0.17% of all cancers in men [2].

The male breast is susceptible to several pathological processes that affect the female breast. The spectrum of male breast diseases, main conventional imaging features of lesions and diagnostic processes are well documented in the literature [1, 2]. Imaging is not required for men with classic gynecomastia or pseudo-gynecomastia. In cases of indeterminate palpable masses, ultrasound (US) is preferred for patients under 25 years of age, while mammography (MX) is reserved for suspicious findings. For men aged 25 years or older or with worrisome clinical features, MX is the initial modality, with US used for further evaluation if the results are unclear or concerning [3]. The indications for breast magnetic resonance imaging (MRI) in women are internationally standardized and supported by established guidelines [4]. However, despite the widespread use of MRI in female breast imaging, its application in the differential diagnosis of male breast disease is limited to a few centers and studies on this topic remain scarce.

When surgery is indicated, MRI can provide valuable information by assessing the posterior extension of the lesion to the chest wall, involvement of the nipple and/or skin, presence of axillary lymph node enlargement and residual post-surgical disease. Additionally, it can help to detect recurrences during follow-up and the response to neoadjuvant chemotherapy [5, 6].

We retrospectively evaluated 58 male breast MRI scans performed at the Radiology Department of the University of Verona between 2017 and 2024. We included only patients who first underwent conventional imaging such as MX and US, and then a US-guided biopsy or surgical excision for histological correlation because of the presence of suspicious findings or unusual presentation, with the exception of a patient affected by breast implant rupture without MRI-detected lesions. Our studies were performed using 1.5 T and 3 T scanners and included STIR/T2-weighted fat-saturated and non-fat-saturated sequences in the axial and coronal planes, axial diffusion-weighted imaging (DWI), and contrast-enhanced 3D T1-weighted fat-saturated (THRIVE or DIXON) images. Post-processing subtraction and Maximum Intensity Projections (MIPs) were also obtained. Morphology and margins, signal appearance, mass or non-mass enhancement and kinetic curves (type 1 or progressive, type 2 or plateau and type 3 or wash-out) were evaluated following the same protocols used for female breast MRI.

The imaging features of various benign and malignant lesions were analyzed and discussed to illustrate how MRI can help radiologists in differential diagnosis, particularly in cases where MX and US findings are inconclusive or point to suspicious lesions. Table 1 summarizes the literature [7] and institutional experience to outline the MRI features of male breast lesions, with comparative insights drawn from female breast imaging owing to the scarcity of dedicated studies. In fact, while certain lesions exhibit similar imaging patterns across sexes, others differ or remain undefined, particularly rare entities such as fibroadenomas and breast metastases.

**Table 1:** Summary of the main features of the most common breast male lesions

Lesion	Nature	Morphologic Findings	T2 WI	DWI	CE	Time–intensity curve
Gynecomastia	Benign	Central–subareolar, with nodular, dendritic or diffuse pattern	Hypointense or possible hyperintense for associated inflammatory component	No obvious restriction	None or low diffuse enhancement (No focal enhancement)	None or Type 1
Lipoma	Benign	Circumscribed mass	Hypointense on fat-suppressed images	No obvious restriction	None	/
Fibroadenoma	Benign	Ovoid–circumscribed mass	Higher signal intensity compared to the surrounding tissue, possible low-intensity internal septations	No obvious restriction	Variable, from avascular to robust and rapid enhancement	None or Type 1
Hemangioma	Benign	Ovoid or lobulated mass	Hyperintense with cavernous cystic spaces containing slow-flowing blood, focal low-intensity foci for calcifications or thrombosis	Low restriction	Progressive enhancement, centripetal in cavernous type, sometimes early and diffuse enhancement (differential diagnosis with angiosarcoma)	Type 1 or 2
Desmoid Tumor	Benign, occasionally locally aggressive	Spiculated large mass	Heterogeneous, low for fibrotic component and high for stromal component	Low restriction	Usually progressive enhancement (from stromal component) also rapid enhancement	Mostly type 1, but also type 2 or 3
Breast Implant Rupture	Benign	Intact or interrupted capsule or other normal findings (calcification, effusion, folding)	Subcapsular line, Droplet, Noose and Keyhole sign, Linguine sign, Extracapsular silicone	No obvious restriction	None or diffuse capsular enhancement	/
Ductal Carcinoma In Situ	Malignant	Calcifications, ductal ectasia, nipple discharge	Possible hyperintense ectasic ducts or hypointense hemorrhagic nipple discharge	Variable, from obvious restricted to no restriction	Non-mass enhancement with a linear or segmental distribution	Type 2 or 3
Invasive Ductal Carcinoma	Malignant	Irregular, spiculated or circumscribed mass, but also indistinct margins	Heterogeneous hyperintense	High restriction	Mass or non-mass enhancement	Type 2 or 3
Invasive Papillary Carcinoma	Malignant	Irregular mass or complex cyst	Heterogeneous hyperintense/hypointense hemorrhagic content	Variable, from low to high restriction	Heterogeneous mass enhancement	Type 1, 2 or 3
Metastases	Malignant	Irregular, spiculated or circumscribed mass, mostly multiple	Heterogeneous hyperintense	High restriction	Mass enhancement	Type 2 or 3

Many cases have been discussed at the Breast Unit Multidisciplinary Meeting (MDM) to establish the most accurate patient care pathway in the presence of members of the Core Team.

The purpose of the study was to provide a review of the literature concerning the main pathologies of the male breast and their MRI appearance and to share a unique and educational collection of images.

## Benign lesions

### Gynecomastia

Gynecomastia is the most common condition affecting the male breast, with a reported prevalence ranging between 33 and 66%, depending on age and diagnostic criteria [8]. Breast hypertrophy is characterized by an increase in breast volume caused by benign proliferation of ductal tissue, glandular stroma or fat. It can be unilateral, bilateral, symmetrical or asymmetrical. Different conditions can lead to gynecomastia, ranging from hormonal imbalances resulting in excess estrogen to obesity, cirrhosis, steroid use, hypogonadism syndrome and kidney failure. Pharmaceutical causes account for up to 20% of all adults [9]. The distinctive clinical trait of gynecomastia is its central and symmetric localization under the nipple [10]. Three patterns of gynecomastia have been described in the literature based on the degree and stage of ductal and stromal proliferation: nodular, dendritic and diffuse [11].

**Fig. 1:**
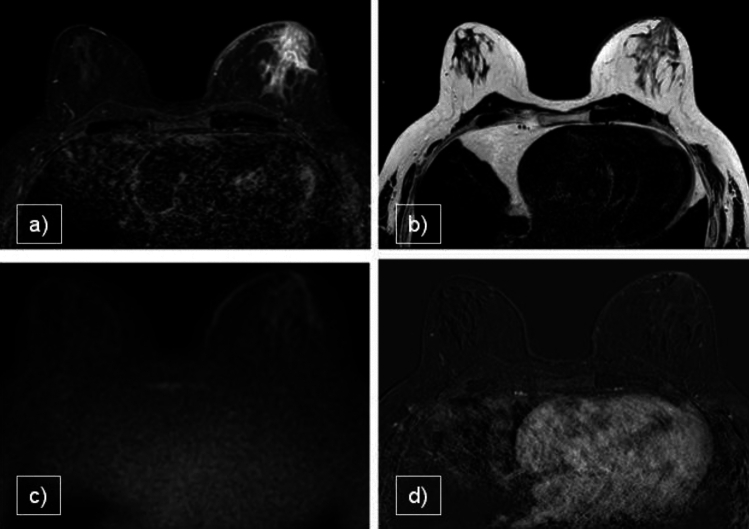
**a** STIR, **b** T2WI VISTA, **c** DWI, **d** CE T1WI. 60-year-old cirrhotic patient with known bilateral breast enlargement and recent episode of tenderness and severe pain on the left side. Bilateral remarkable gynecomastia associated with left mastitis (histological confirmation was obtained for precautionary purpose). On MRI thickness of the skin and edema of the glandular tissue is evident on the left breast in comparison with the contralateral. MRI excluded malignancies both with DWI and CE images. Topical treatment was performed and follow-up suggested

The gold standard for the diagnosis of gynecomastia is MX followed by US in adults, as they both have high sensitivity and specificity [12], while only US is used in children. In cases with an atypical presentation, biopsy may be necessary to differentiate gynecomastia from malignancy. On MRI, gynecomastia is characterized by subareolar glandular tissue that is hypointense on T1-weighted (T1WI) and T2-weighted (T2WI) images, with no restriction on DWI. Gynecomastia can also present with an associated inflammatory component that alters the glandular parenchyma and adjacent adipose tissue, leading to changes in the signal intensity across various pre-contrast sequences. In such cases, administration of a contrast agent is invaluable in excluding the presence of malignancy, as gynecomastia never exhibits areas of focal enhancement (Fig. 1).

### Lipoma

Lipomas are the most common benign tumors of the male breast and are composed of mature fat cells. It manifests as a soft, nontender, palpable subcutaneous mass.

A lipoma appears on MX as a fat density lesion that is indistinguishable from the adjacent fat. This corresponds to a mildly hyperechoic, circumscribed, oval mass on US. Owing to its typical imaging features, this lesion does not require a biopsy. Some patients choose elective surgical excision when the lesion is cosmetically unacceptable [13, 14].

**Fig. 2:**
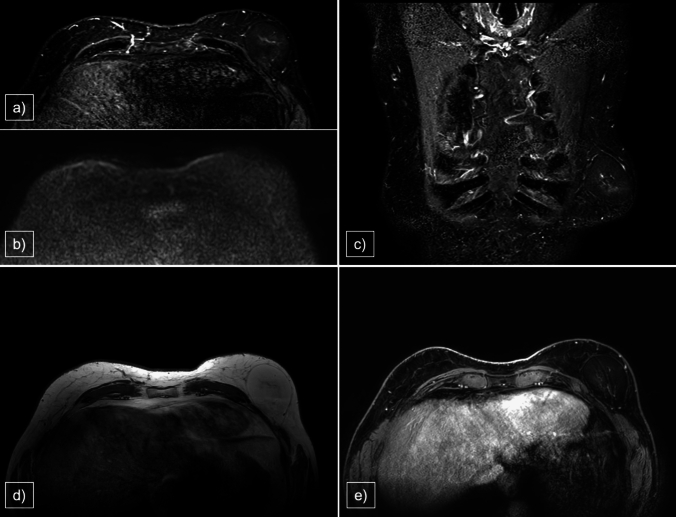
**a** STIR ax, **b** DWI, **c** STIR cor, **d** T1WI, **e** CE T1WI. A 66-year-old male patient referred a slow progressive soft lump on left breast, without pain. A 7-cm-capsulated fat lesion was present in the outer quadrants. MRI was performed because of the considerable size and for surgical planning. It appeared hyperintense on T1WI and T2WI, with low intensity on STIR and after fat saturation on post-contrastographic images. No enhancement was present, excluding malignancy. Surgical excision was executed and a benign lipoma was histologically confirmed

The MRI features of lipoma include a circumscribed mass with a hyperintense fat signal on T1WI and homogeneous suppression on fat-saturated images, with no enhancement after gadolinium injection or restriction on DWI sequences (Fig. 2). These characteristics, particularly the absence of thickened and vascularized branches, enable us to exclude liposarcoma as the primary differential diagnosis for lipoma [15].

### Fibroadenoma

Fibroepithelial lesions are uncommon in the male breast. Fibroadenomas typically manifest in men with elevated levels of estrogen and progesterone and are often seen in medical conditions such as prostate carcinoma or in transgender patients, owing to the presence of hormone receptors in these lesions. Fibroadenomas are exceedingly rare in healthy men because of the absence of lobular structures, even in the presence of gynecomastia [16, 17]. We did not find any documented examples of fibroadenomas in our case series. However, the imaging characteristics of this lesion can readily be correlated with those observed in the female breast.

On MX, the mass resembled a female fibroadenoma, presenting as a circumscribed, isodense/slightly hyperdense structure relative to the fibroglandular tissue, with lobulated margins or calcification. On US, fibroadenomas appear as homogeneously hypoechoic masses with no significant internal vascularization on color Doppler US [16]. On MRI, fibroadenomas are typically well-circumscribed masses with variable signal intensities on T2WI based on the fibrotic matrix of the lesion. Low-signal-intensity internal septations, characteristic of fibroadenomas, may be observed and best visualized on non-subtracted post-contrast images or T2WI. Similar to female fibroadenomas, those in males exhibit variable enhancement patterns on dynamic contrast-enhanced MRI, ranging from avascular to robust and rapid enhancement. Most fibroadenomas display a progressive enhancement curve when analyzing the signal intensity time course, whereas wash-out patterns are rare [18].

### Hemangioma

Breast hemangiomas are rare benign vascular tumors with an incidence of 11% in postmortem female breast specimens [19]. Owing to its rarity, data on its occurrence in men are limited to a few case reports [20]. They are the result of neoangiogenesis, which is histologically characterized by vascular channels filled with erythrocytes. They can occur both within and beyond lobular units and are classified as capillary or cavernous [19].

Hemangiomas appear as oval or lobulated superficial masses with circumscribed margins and a density similar to that of fibroglandular breast tissue [19]. Occasionally, fine calcifications may be observed on MX. On US, their appearance may vary from an ill-defined hypoechoic mass to a hypoechoic or cystic area with small bright echoes owing to calcification or fibrous septations [21]. Because of these features, differentiating hemangiomas from fibroadenomas or complex cysts may be challenging. In suspicious cases where MX or US features are atypical, it is crucial to exclude malignant lesions, such as angiosarcoma, although extremely rare, and ductal carcinoma in situ (DCIS) due to fine calcifications. In such cases, biopsy is required and MRI may be useful.

**Fig. 3:**
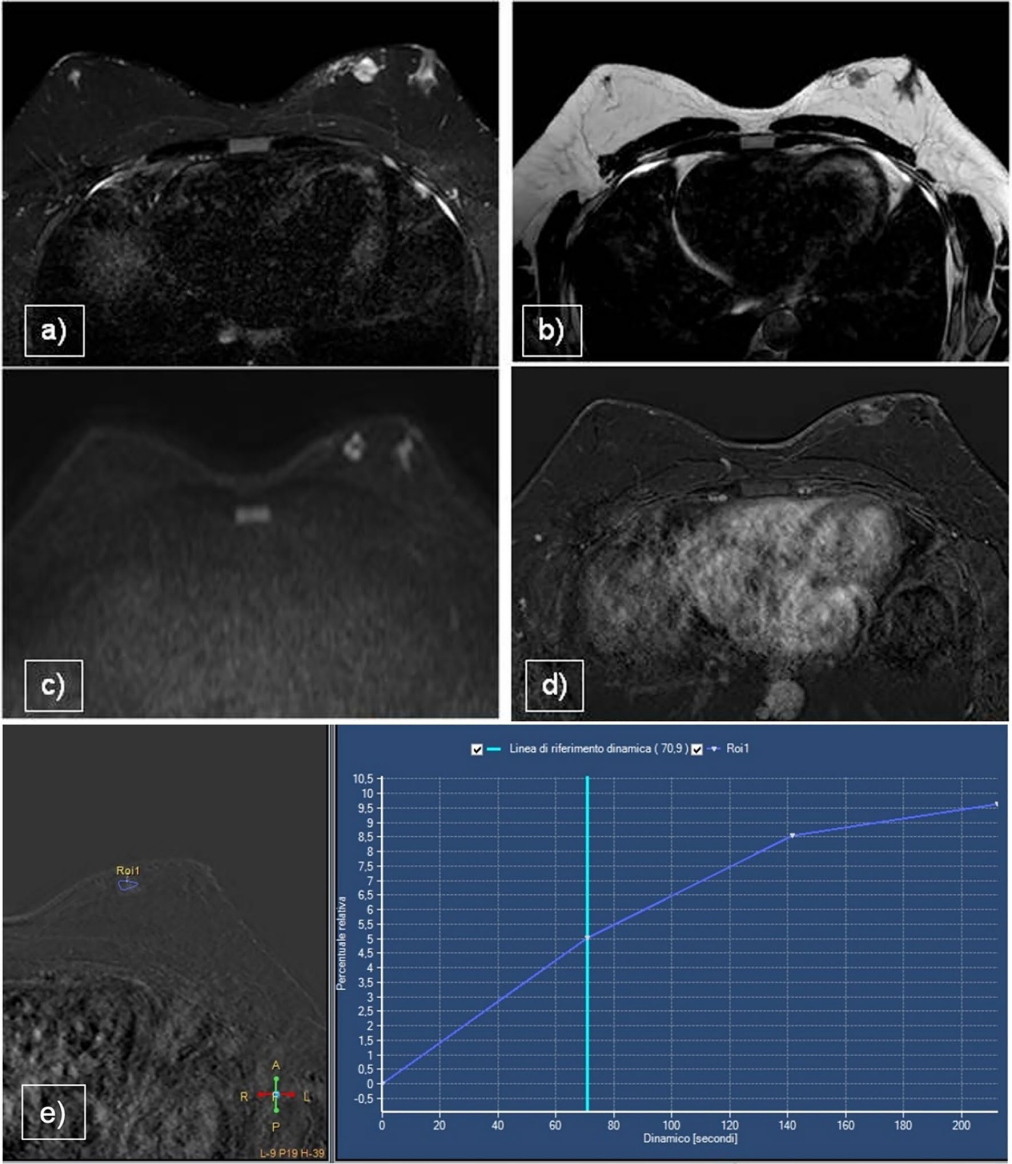
**a** STIR, **b** T2WI, **c** DWI, **d** CE T1WI, **e** Time–Intensity Curve. 52-year-old man with a small hard lump appearing between the inner quadrants of the left breast, biopsy was performed with a diagnosis of hemangioma. The vascular tumor demonstrated capsulated margins, DWI restriction and a progressive enhancement (kinetic curve type 1)

MRI findings of hemangiomas typically show ovoid or lobulated masses, hypointense to fibroglandular tissue on T1WI, hyperintense on T2WI and STIR, cavernous cystic spaces containing slow-flowing blood and focal low-intensity foci owing to the presence of calcifications or thrombosis. DWI sequences revealed mild restriction. On contrast-enhanced T1WI fat-sat subtraction sequences, hemangiomas exhibited progressive enhancement, centripetal in the cavernous type, with a type 1 curve (Fig. 3). In some cases, hemangiomas may present with early and diffuse enhancement due to the presence of numerous vascular channels, making it difficult to differentiate hemangiomas from angiosarcomas [22].

### Desmoid tumor

Desmoid tumor (fibromatosis) is an uncommon benign stromal tumor that arises from the proliferation of fibroblasts and myofibroblasts within the breast parenchyma and accounts for less than 0.2% of all breast tumors [23]. It may range from inert to locally aggressive but without metastatic potential. However, they occurred sporadically (97% of cases). They could follow trauma, surgery or occur in specific conditions, such as Gardner Syndrome, adenomatous polyposis and familial multicentric fibromatosis. Most cases of desmoid tumors occur in women (3:1), with only a few cases reported in men [2]. These tumors usually appear as large masses, ranging from 0.5 to 10 cm at presentation.

Desmoid tumors appear as spiculated masses without calcification on MX, whereas on US, they are hypoechoic with irregular or lobulated margins. In most cases, they do not exhibit posterior shadowing or hyperechoic halo. Nipple discharge or axillary lymphadenopathy is uncommon and its presence suggests a malignancy instead of a desmoid. Biopsy is required to make a diagnosis, but MRI can be useful to evaluate the extent of the tumor, its relationship with neurovascular structures and chest wall involvement, thus playing an essential role in pre-surgical planning. Therefore, surgical resection with negative margins is recommended. The recurrence rate after surgery ranged from 18 to 29% [24].

**Fig. 4:**
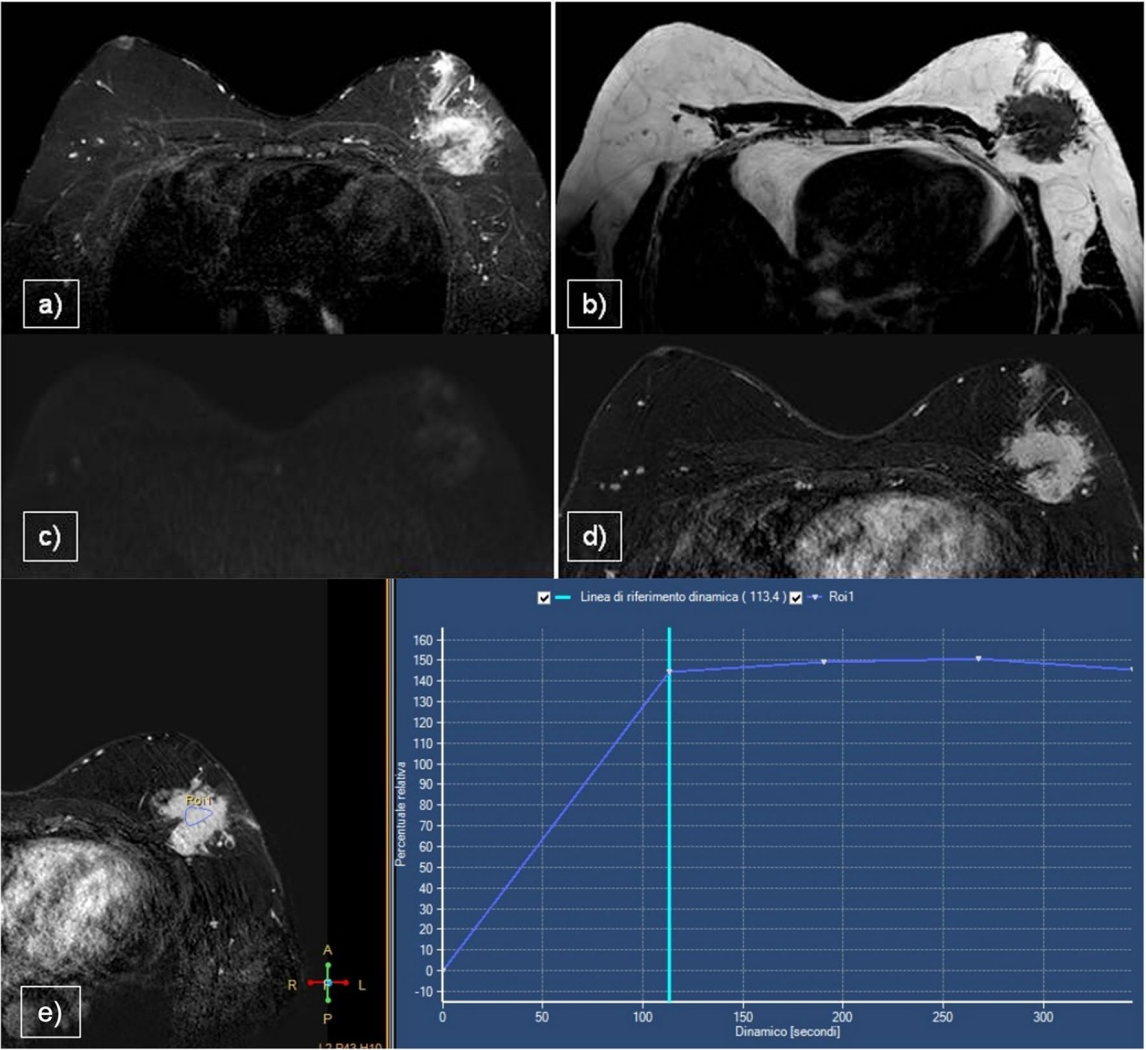
**a** STIR, **b** T2WI, **c** DWI, **d** CE T1WI, **e** Time–Intensity Curve. A 6-cm hard lesion is observed on the left breast, consisting of two different tissue components: a hyperintense stromal component in STIR and a hypointense fibrous component in all sequences (**a**,**b**). The mass demonstrates low-signal restriction in DWI (**c**), intense ehancement (**d**) and a plateau time–intensity curve (**e**)

On MRI, desmoid tumors appear as large masses that are isointense to muscle on T1WI, heterogeneous with low to hyperintense signals on T2WI (low for the fibrotic component and high for the stromal component) and show low restriction on DWI. These characteristics reflect both the increased cellularity and dense fibrosis. After intravenous administration of contrast medium, desmoid tumors show strong enhancement, usually with a progressive kinetic curve, although plateau or washout kinetics have been described in the literature [25]. Contrast-enhanced imaging also clearly shows invasion of the pectoralis muscle and extent of involvement of the entire chest wall (Fig. 4). The lesion described in our case study underwent surgery and recurrences were managed using percutaneous cryoablation. Desmoid fibromatosis is a highly aggressive lesion with a high recurrence rate, particularly in extra-abdominal locations and often benefits from minimally invasive treatments. Percutaneous cryoablation has demonstrated efficacy as an alternative to surgical intervention, offering a less invasive approach with comparable outcomes [26].

### Breast implant rupture in MtF transgender patients

It is essential to study and screen Male-to-Female (MtF) and Female-to-Male (FtM) individuals because of their distinct cosmetic complications and cancer risks. Radiology plays a key role in providing inclusive and effective care to transgender patients.

**Fig. 5:**
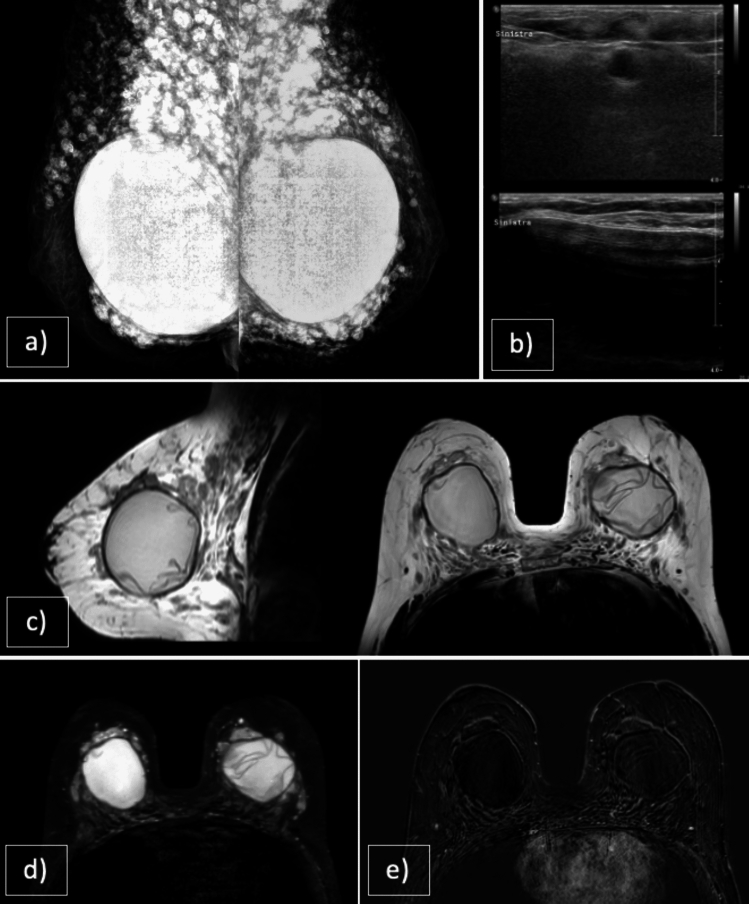
**a** MX, **b** US, **c** sagittal and axial T2WI, **d** silicon-only sequence and **e** CE T1WI. Breast augmentation with silicone implants and several injection of silicone material was performed in a 40-year-old MtF patient. MX and US reveal extensive coverage of the prosthetic implants by the silicone injection, which appears radiopaque on MX and produces a diffuse *snowstorm-like* effect on US. To further assess the patient, an MRI was conducted, which clearly demonstrated an intracapsular rupture of the left implant, characterized by the *linguine sign*. No pathological enhancement was observed, excluding an otherwise invisible malignancy. The patient was entrusted to the plastic surgeon

Breast implant ruptures in MtF are relatively under-researched, as most studies have focused on female populations. However, men who receive breast implants, primarily for sex affirmation surgery or cosmetic correction, may experience similar complications, including implant rupture. Implant damage can present with symptoms, such as chest pain, changes in chest contour or the presence of nodules or hardening in the implant area. However, as in female patients, many ruptures are silent, meaning that they occur without noticeable symptoms, necessitating the use of advanced imaging techniques for accurate diagnosis [27]. Radiology plays a crucial role in the diagnosis and management of implant ruptures by providing detailed imaging to evaluate the extent of the damage and assist in treatment planning. This is particularly relevant in cases involving non-standard procedures, such as extra-prosthetic silicone injections, which substantially increase the diagnostic complexity. Effective evaluation in these scenarios necessitates the judicious use of all available imaging modalities to ensure accurate characterization, optimal management and challenges that radiologists are likely to encounter throughout their clinical practice (Fig. 5).

US is frequently used as the first diagnostic tool. It can detect findings like the *snowstorm sign,* indicative of extracapsular silicone dispersion into surrounding tissues [28]. The unique anatomy of the male chest, with different soft tissue distributions and types of surgical interventions, may influence US imaging and MX and requires adjustment of the technique. MX can help detect capsular calcifications or gross alterations in implant shape.

MRI is the gold standard for detecting intracapsular and extracapsular ruptures. Key indicators of silicone implant rupture include subcapsular and hypointense lines parallel to the fibrous capsule. When these lines are paired and aligned with the capsule, they create the *railroad track* sign, an early form of the *linguine sign*. Another important finding is the presence of free silicone or silicone granulomas, which indicate a breach of the implant shell and capsule, leading to silicone leakage into the surrounding tissues. The *linguine sign* characterized by low-signal-intensity curved lines within the silicone, represents collapsed elastomer contained by the fibrous capsule, the most sensitive and specific marker of rupture. Furthermore, the detection of free silicone in the breast tissue or axillary lymph nodes, often in combination with intracapsular rupture, is the most reliable sign of extracapsular rupture [29].

Once a rupture is confirmed, its management depends on the nature of the rupture and the patient’s symptoms. Extracapsular rupture and symptomatic cases typically require implant removal or replacement. Silent intracapsular ruptures should be monitored; however, regular radiological follow-up is essential.

Due to its rarity but its clinical relevance, the identification of breast cancer in transgender individuals is an emerging topic in the literature [30–32]. MtF people undergoing long-term estrogen-based hormone therapy may develop estrogen receptor–positive (ER +) breast tumors, with 21 non-implant-associated breast cancer cases reported since 1968, mostly invasive carcinomas. Although the overall incidence in transgender women is still lower than that in cisgender women, those with BRCA1/2 mutations or extended hormone exposure face an increased risk compared to cisgender men. In FtM individuals, the lifetime breast cancer risk remains 12.4%, similar to that in cisgender women, unless a bilateral mastectomy is performed which reduces the risk by nearly 90%. Nevertheless, the risk still exceeds that of cisgender men. The incidence rate is 5.9/100,000 person-years in FtM compared to 154.7 in cisgender women, underscoring the need for individualized, risk-aware screening strategies using advanced imaging when appropriate.

## Malignant lesions

Male breast cancer accounts for approximately 0.7% of all breast cancers and 0.17% of all cancers in men [1, 2, 33]. The mean age at diagnosis is 59 years, 6–11 years later than that of the female population. In men, the prognosis is usually worse [2], possibly linked to a diagnostic delay caused by the absence of mammographic screening. The main risk factors for the development of male breast cancer are BRCA1 and BRCA2 mutations, positive family history of breast cancer, Klinefelter syndrome, hyperestrogenism, advanced age and history of chest irradiation [2, 34].

Since males have only breast duct tissue without terminal lobes, invasive ductal carcinoma (IDC) is the most frequent histologic type, representing 85% of all male breast cancer cases, followed by DCIS occurring in 5% of cases and invasive papillary carcinoma in 2% [35]. It should be noted that while invasive lobular carcinoma represents 10% of breast tumors in women [36], in men it is a rare condition, along with other less common subtypes with mixed features [14].

### Ductal carcinoma in situ

Male DCIS patients may present with symptoms, such as a palpable mass or bloody nipple discharge. MX examination may reveal pleomorphic microcalcifications, which are small calcium deposits within breast ducts. If left untreated, DCIS can progress into invasive breast cancer. Studies have shown that within 10–20 years, approximately 30–50% of untreated DCIS cases may develop into invasive cancer. Therefore, early detection and treatment of DCIS are crucial to prevent its progression to invasive disease [35], which is particularly relevant in the male population where no routine screening programs are available for the timely identification of breast cancer.

**Fig. 6:**
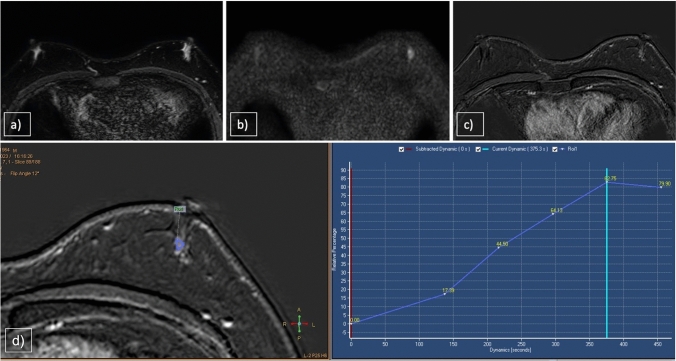
**a** STIR, **b** DWI, **c** CE T1WI, **d** Time–Intensity Curve. 32-year-old patient with bloody nipple discharge on the left. The MRI showed bilateral gynecomastia with restricted signal in DWI on the left (**a**,**b**,**c**). Subareolar non-mass enhancement with a type 1 time–intensity curve was evident (**d**). Biopsy confirmed malignant nature of the lesion (DCIS)

In our experience, the MRI features of DCIS include non-mass enhancement with ductal distribution and variable kinetics from early washout to persistent enhancement (Fig. 6).

### Invasive ductal carcinoma

Clinically, IDC manifests as a painless palpable mass in the breast, typically unilateral and located eccentrically to the nipple. It may also be associated with gynecomastia. Involvement of the nipple-areola complex is frequent with symptoms such as retraction, ulceration or nipple discharge. In almost half of the cases, IDC was associated with DCIS [37].

On MX, IDC appears as a high-density mass with irregular, spiculated, lobulated or microlobulated margins. Microcalcifications are uncommon and occur in only a small percentage of cases (13–30%). A non-parallel hypoechoic solid mass with poorly defined margins is typically identified using US. Posterior acoustic shadowing may be present, but is not exclusive to malignant lesions. A color Doppler study may be useful for demonstrating the presence of vascularization.

When exploring the axillary regions, pathological lymphadenopathy with loss of cortical–medullary differentiation was found in 50% of cases [38]. The identification of these node findings invariably necessitates further evaluation through fine-needle aspiration or, when appropriate, core biopsy.

**Fig. 7:**
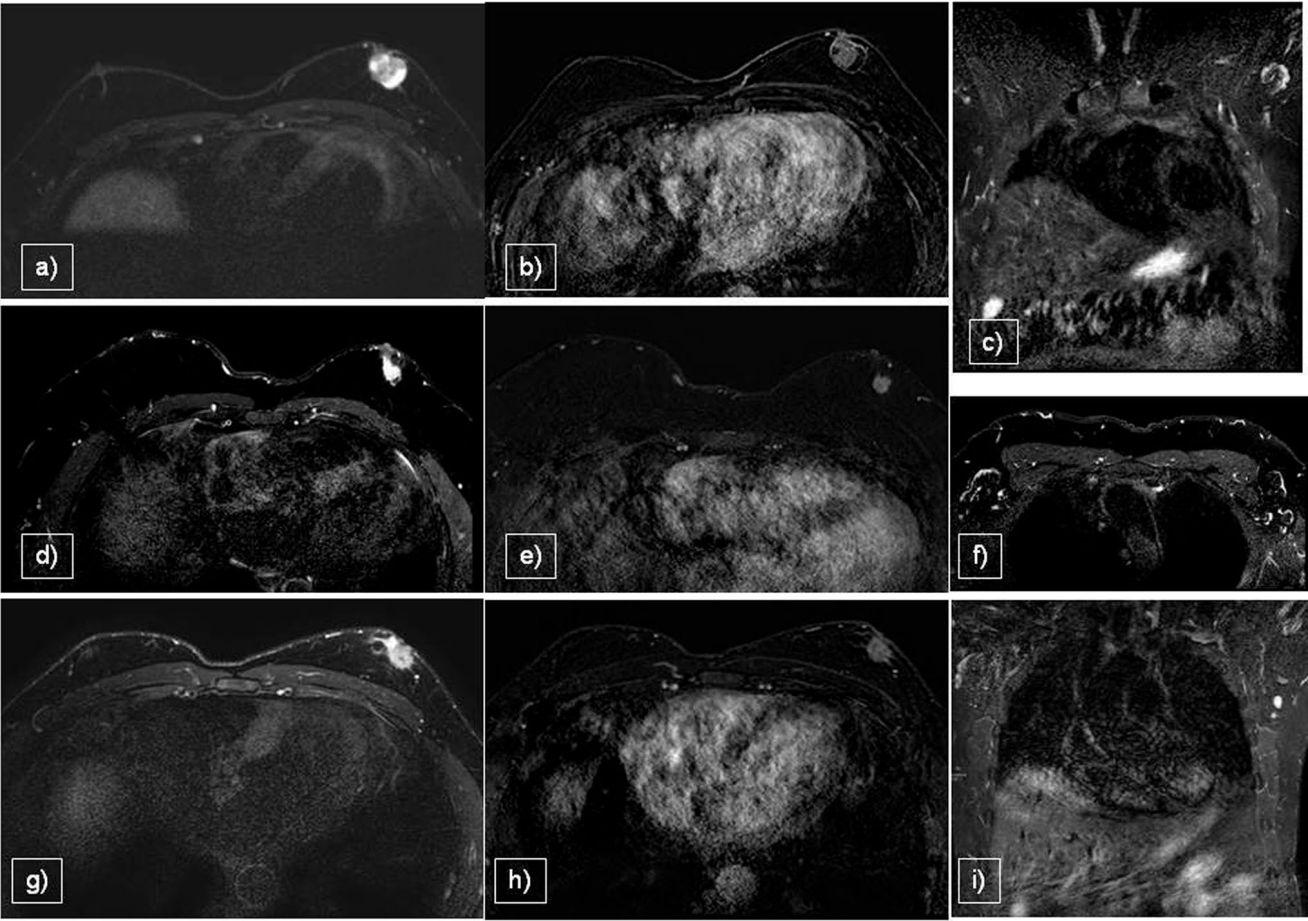
Papillary carcinoma **a**,**b**,**c**, (IDC) G2 **d**,**e**,**f**, and IDC G3 **g**,**h**,**i** presented in three sequences: STIR, CE T1WI and STIR for axillary evaluation. MRI demonstrated lesions with irregular margins and enhancement, nipple/skin infiltration (**b**,**h**), and node involvement seen only in IDC G3 (**i**)

**Fig. 8:**
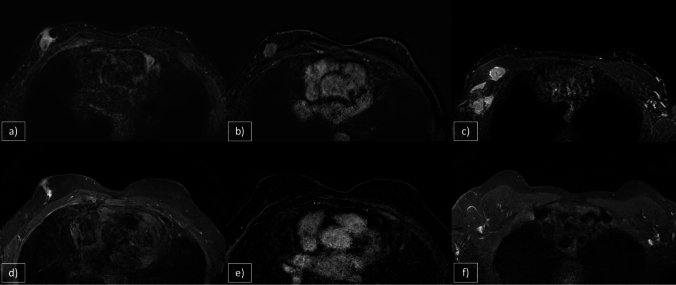
Male patient presented with bilateral gynecomastia and IDC on the right, deemed a candidate for neoadjuvant chemotherapy. Staging breast MRI revealed a hyperintense lesion on STIR sequences (**a**) and early enhancement on T1-CE images (**b**), accompanied by multiple ipsilateral axillary and retropectoral lymphadenopathies (**c**). Following six months of neoadjuvant chemotherapy, the patient demonstrated an excellent therapeutic response, (**d**) characterized by the absence of mass enhancement on post-contrast imaging (**e**). Additionally, no residual axillary lymphadenopathies were identified (**f**)

In patients with IDC (Fig. 7d–i), MRI is required to evaluate the size of the lesion in pre-surgical planning, especially the posterior extent, chest wall involvement and distance from the areolar–nipple complex. Moreover, MRI may be used to evaluate post-neoadjuvant treatment (Fig. 8) and post-surgical residual disease, and for the follow-up and screening of high-risk patients in conjunction with MX and US. On T1WI, IDC lesions typically appear as irregular or circumscribed hypointense masses, reflecting the dense fibrous nature of the tumor and its low signal intensity relative to the surrounding adipose and glandular tissue. In contrast, on T2WI, these lesions exhibit hyperintensity, indicative of increased water content associated with tumor-related edema, necrosis and high cellularity, which is a common feature of malignant tumors. On DWI, IDC lesions typically exhibit high restriction and low ADC values. After the administration of paramagnetic contrast agent, IDC lesions typically exhibit intense and homogeneous enhancement, often presenting as a well-defined mass or non-mass enhancement. IDC lesions usually display a plateau or washout pattern in kinetics curves [6].

### Invasive papillary carcinoma

Invasive papillary carcinomas are uncommon male tumors, representing 2% of all histopathological subtypes of male breast cancers [37]. They belong to a heterogeneous group of papillary carcinomas, which also include intraductal papillomas with or without atypia and noninvasive papillomas (in situ). When explored using MX, they usually appear as subareolar masses, with well-defined or spiculated margins. Rare is the occurrence of calcifications. US indicates that both invasive and in situ papillary lesions typically manifest as complex cysts with mixed solid and cystic morphological features, often accompanied by cystic formations, hemorrhagic phenomena or dilated ducts. When a complex cystic mass is recognized in men, biopsy is strongly recommended [1].

MRI findings of papillary carcinomas are limited to a few case reports [38] and their relevance is associated with preoperative planning for surgical cases, particularly for the resection of multiple lesions [39]. MRI (Fig. 7a–c) revealed papillary carcinomas as irregular nodules or complex cysts with heterogeneous contrast enhancement. The kinetic curves were variable [40].

### Metastases

Secondary breast tumors from non-mammary malignancies are uncommon, accounting for 0.5% to 3% of all breast tumors, with only 5% occurring in male patients. The most frequent primary cancers that metastasize to the breast include melanoma, non-Hodgkin lymphoma, lung carcinoma, sarcomas, and malignancies of the stomach, kidney, prostate and ovaries [41]. Hematogenous spread usually results in well-defined, localized masses, whereas lymphatic dissemination often presents as diffuse lesions resembling inflammatory breast conditions [42].

**Fig. 9:**
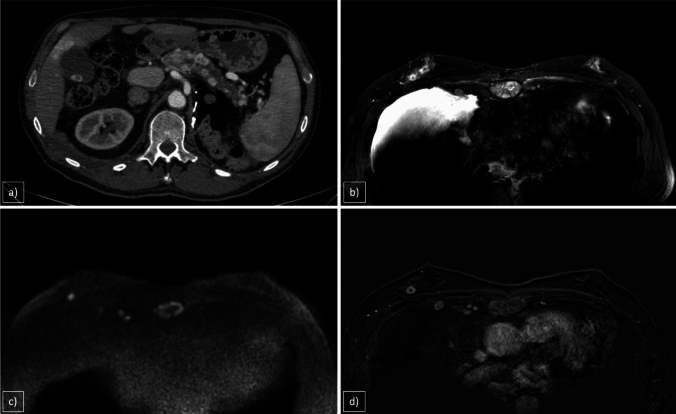
**a** Arterial Phase CT scan, **b** STIR, **c** DWI, **d** CE T1WI. 78-year-old man with a history of left nephrectomy for renal carcinoma and multiple pancreatic metastases. The patient complains about the appearance of a palpable solid nodule in the right breast. MRI reveals a solid lesion of 10 mm with central necrosis on STIR sequences, accompanied by pleural effusion and pleural nodules. These pleural nodules demonstrate restricted diffusion and post-contrast rim-enhancement, along with evidence of bone remodeling at the sternal level. An US-guided biopsy confirmed the diagnosis of renal carcinoma metastasis

On MX, these lesions typically appear as round, high-density masses with smooth, well-defined borders, resembling benign entities. Unlike primary breast tumors, metastatic lesions lack irregular or spiculated edges and do not cause nipple or skin retractions. Calcifications, although uncommon, may be associated with metastases from ovarian, hepatocellular, medullary thyroid or gastric cancer [43]. US often reveals metastatic lesions as round masses with distinct or slightly indistinct borders. Most are hypoechoic with occasional hyperechoic or anechoic areas. Unlike primary breast tumors, these lesions rarely exhibit architectural distortions or posterior acoustic shadowing. Color Doppler can help differentiate malignant from benign lesions, whereas pathologic axillary lymphadenopathy is less frequently observed in metastatic cases [41–43]. On MRI, metastatic breast lesions generally appear as sharply demarcated masses with low signal intensity on T1WI, intermediate signal intensity on T2WI or a strong diffusion restriction on DWI. After administration of contrast media, these lesions often show homogenous/heterogeneous intense enhancement, mimicking the behavior of their primary tumors. The enhancement kinetics frequently demonstrated plateau or washout patterns in delayed imaging (Fig. 9).

## Limitations

MRI plays an important role in evaluating male breast conditions but has several limitations that should be considered. A key issue is the risk of false positives, especially in cases where benign conditions such as gynecomastia resemble malignancies on imaging. However, MRI can also produce false negatives, particularly when dealing with small lesions or those that do not form a distinct mass, potentially resulting in missed diagnosis. The interpretation of MRI findings can vary significantly between institutions and is influenced by differences in imaging protocols, equipment and contrast agent use. Additionally, the accuracy of MRI is closely tied to the radiologist’s level of expertise and familiarity with male breast anatomy, which may be limited due to the infrequent occurrence of such cases. These challenges highlight the need for consistent imaging standards, increased training and collaborative diagnostic efforts to enhance the reliability of MRI in the assessment of male breast pathology.

## Conclusions

Male breast cancer remains a relatively rare and under-researched entity with limited data guiding its diagnosis and management. Notably, it is associated with lower survival outcomes than breast cancer in women, largely due to delayed diagnosis and a lack of standardized imaging protocols. Current guidelines from the European Society of Breast Imaging and the American College of Radiology do not recommend MRI as a routine tool for the evaluation of male breast lesions [44, 45]. Nevertheless, emerging evidence suggests that MRI may play an important adjunctive role in certain clinical contexts.

MRI offers superior soft-tissue contrast and functional imaging capabilities, enabling improved delineation of lesion extent, internal architecture and tissue characteristics through DWI and dynamic contrast-enhanced sequences. In particular, MRI can be beneficial in patients with complex or indeterminate findings on MX or US as well as in transgender individuals with breast implants, where it remains the modality of choice for detecting both intracapsular and extracapsular implant ruptures.

Furthermore, MRI is instrumental in assessing local invasion, including the nipple-areolar complex or skin involvement and estimating the lesion-to-nipple distance. It can also provide valuable information on the regional lymph node status, which is critical for staging and therapeutic planning. In cases of atypical lesion presentation or discordant imaging findings, MRI can assist in lesion characterization and US-targeted biopsy. MRI is the best tool for evaluating treatment efficacy during neoadjuvant chemotherapy.

Given the distinct anatomical and pathological features of male breast cancer, there is an urgent need to develop dedicated diagnostic pathways and imaging guidelines. Future research should focus on optimizing imaging protocols and validating the clinical utility of MRI in this population to improve the diagnostic accuracy and patient outcomes.
